# Dysbiosis in gastrointestinal pathophysiology: Role of the gut microbiome in Gulf War Illness

**DOI:** 10.1111/jcmm.17631

**Published:** 2023-01-30

**Authors:** Elise Slevin, Sachiko Koyama, Kelly Harrison, Ying Wan, James E. Klaunig, Chaodong Wu, Ashok K. Shetty, Fanyin Meng

**Affiliations:** ^1^ Division of Gastroenterology and Hepatology, Department of Medicine Indiana University School of Medicine Indianapolis Indiana USA; ^2^ Richard L. Roudebush VA Medical Center Indianapolis Indiana USA; ^3^ Department of Transplant Surgery Baylor Scott & White Memorial Hospital Temple Texas USA; ^4^ Department of Pathophysiology, School of Basic Medical Science Southwest Medical University Luzhou China; ^5^ Laboratory of Investigative Toxicology and Pathology, Department of Environmental and Occupational Health, Indiana School of Public Health Indiana University Bloomington Indiana USA; ^6^ Department of Nutrition Texas A&M University College Station Texas USA; ^7^ Department of Molecular and Cellular Medicine Institute for Regenerative Medicine, Texas A&M College of Medicine College Station Texas USA

**Keywords:** extracellular vesicles, Gulf War Illness, gut microbiome, high‐fat diet, microRNAs

## Abstract

Gulf War Illness (GWI) has been reported in 25%–35% of veterans returned from the Gulf war. Symptoms of GWI are varied and include both neurological and gastrointestinal symptoms as well as chronic fatigue. Development of GWI has been associated with chemical exposure particularly with exposure to pyridostigmine bromide (PB) and permethrin. Recent studies have found that the pathology of GWI is connected to changes in the gut microbiota, that is the gut dysbiosis. In studies using animal models, the exposure to PB and permethrin resulted in similar changes in the gut microbiome as these found in GW veterans with GWI. Studies using animal models have also shown that phytochemicals like curcumin are beneficial in reducing the symptoms and that the extracellular vesicles (EV) released from gut bacteria and from the intestinal epithelium can both promote diseases and suppress diseases through the intercellular communication mechanisms. The intestinal epithelium cells produce EVs and these EVs of intestinal epithelium origin are found to suppress inflammatory bowel disease severity, suggesting the benefits of utilizing EV in treatments. On the contrary, EV from the plasma of septic mice enhanced the level of proinflammatory cytokines in vitro and neutrophils and macrophages in vivo, suggesting differences in the EV depending on the types of cells they were originated and/or influences of environmental changes*.* These studies suggest that targeting the EV that specifically have positive influences may become a new therapeutic strategy in the treatment of veterans with GWI.

## 1 INTRODUCTION

**FIGURE 1:**
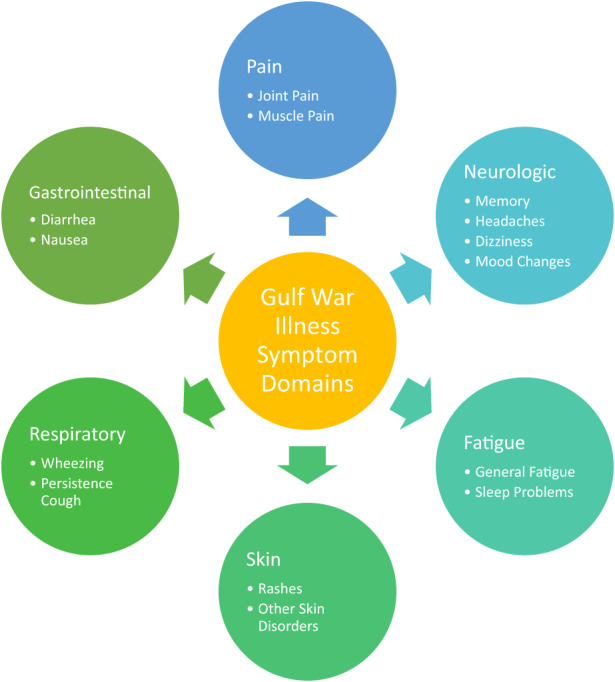
Areas of Gulf War Illness (GWI) symptoms. Symptoms of GWI are broad in range, including gastrointestinal (GI) problems, fatigue, headaches, respiratory and dermatological problems as just few of the examples. Additionally, there is a wide variety of neurological symptoms including trouble with cognitive function, concentration, depression and anxiety.

Following the Gulf War, many GW veterans had unexplained chronic health conditions, which came to be known as Gulf War Illness (GWI).^1^ Approximately, 25%–35% of veterans from the Gulf War would show symptoms of GWI.^1^, ^2^, ^3^ Symptoms are broad in range, including gastrointestinal (GI) problems, fatigue, headaches, respiratory and dermatological problems as just few of the examples^1^, ^4^, ^5^ (Figure 1). Additionally, there is a wide variety of neurological symptoms including trouble with cognitive function, concentration, depression and anxiety.^1^, ^4^ Female veterans have a significantly higher rate of GWI than their male counterparts^6^ as well as symptoms specific to females.^7^, ^8^ Among the broad range of the symptoms, the gastrointestinal symptoms such as diarrhoea, nausea and irritable bowel syndrome were a major group of symptoms reported by the GWI veterans.^4^, ^9^, ^10^, ^11^ Even the most commonly reported symptoms, that is chronic fatigue, can also be associated with changes in the gut microbiome.^12^, ^13^ Thus, the gastrointestinal system and the gut microbiota can have a significant role in GWI, and the understanding of their roles and the development of treatments targeted to the gastrointestinal system may unravel a breakthrough in treating GWI.

Veterans of the Gulf War had a long list of chemical exposures including sarin/cyclosarin and mustard gas, pesticides, depleted uranium, hexavalent chromium and solvents such as benzene.^2^ Investigations into the cause of GWI have shown that chemical agents particularly PB, which was used in pre‐treatment for nerve agents, and permethrin, a pesticide used during the Gulf War, likely played a major role in the development of the illness.^2^, ^10^ Recent studies have shown that chemical exposure alters gut microbiome profile and causes gut dysbiosis,^14^, ^15^ suggesting the importance of studying the gut microbiota in relation to GWI.

Another important development in the recent studies is the finding about the roles of the extracellular vesicles (EV) released from the bacteria in the gut in causing the symptoms of GWI. GW veterans with GWI showed decrease in the gut microbiota (dysbiosis) and alteration in the bacteria species profile.^11^, ^16^, ^17^ Some of the symptoms of GWI could be caused by the dysbiosis and the alteration. EV from the bacteria are found to have a significant role in gut–brain communication,^18^ and changes in the gut microbiota may result in qualitative and quantitative changes in the EV, which will thus in turn cause changes in the gut–brain communication. This suggests a possibility that there might be multiple factors involved in the GWI symptoms caused by the changes in the gastrointestinal system, that is dysbiosis, the alteration in the bacteria species profile, and the change in the EV released from bacteria because of these changes in the gut microbiota.

Here, we will briefly summarize the GWI, the changes in the gut microbiota in diseases, the roles of EV released from gut microbiota in gut–brain communication, and the future possible directions of the studies on the EV from gut microbiota as a potential target in the treatment of GWI.

## 2 THE SYMPTOMS AND AETIOLOGY OF GWI

A meta‐analysis of 21 studies on the self‐reported symptoms of GWI has found that reports on irritability, feeling of detached, muscle weakness, diarrhoea and rash were more than three times higher than the control veterans of around the same age.^4^ Symptoms range from these related to neurologic/mood/cognition, pain, respiratory, musculoskeletal, gastrointestinal and dermatological system, to overall fatigue.^4^, ^19^ Although the range of symptoms reported is broad and diverse, gastrointestinal symptoms are highly prevalent especially in the veterans of GW.^11^, ^20^ Diarrhoea was reported by 10%–27% in the GW veterans compared to only 2%–3% in nondeployed troops,^21^ indicating the extremely high prevalence of gastrointestinal problems in the GW veterans. Abdominal pain, nausea and loose bowel movements were also extremely high in the GW veterans compared to the nondeployed individuals (70%, 23% and 74%, respectively, in the GW veterans, compared to 9%, 2% and 18%, respectively, in the nondeployed veterans).^21^

There are many types of gastrointestinal symptoms reported by GWI patients, for example, diarrhoea, abdominal pain, irritable bowel symptoms and intestinal hyperpermeability.^11^, ^21^, ^22^ Zhang et al.^22^ have shown that veterans with gastrointestinal symptoms have high intestinal permeability, and the increased intestinal permeability correlated with the rating on abdominal pain and other gastrointestinal symptoms. Kimono^11^ discusses that there are three major mechanisms involved causing the inflammation observed in veterans with GWI, which are (1) compromised gut integrity, (2) dysfunctional enteric nervous system and (3) altered microbiome resulting from chemical exposures and war stress.

Gut integrity is an expression about the overall intactness of the intestine or how healthy the gut is. Once a person gets illness, the apoptosis increases in the intestinal epithelial cells, and the cell proliferation/migration and turnover in the intestine, which takes place weekly in the normal condition, decrease.^23^ Such increase of apoptosis and decrease in the turnover can explain some of the reasons of the malfunctioning in the gastrointestinal system. The hyperpermeability of the gut system, that is the reduced ability in its function as a barrier, can be considered one aspect of reduced gut integrity and can lead to a damage in other tissues and organs at distant locations in the body.

The enteric nervous system is in charge of regulating the secretion, motility, mucosal maintenance and immunological defence of the gastrointestinal system.^24^ It communicates with the CNS in a bidirectional way that CNS affects the enteric nervous system, and the enteric nervous system sends information at the gut to the brain that stimulates, for example, nausea and satiety.^24^ Malfunctioning in the enteric nervous system can cause decreased gastrointestinal functioning. From the larger number of afferent fibres from gut towards the brain, the communication is considered more in a gut‐to‐brain direction rather than the other way round.^24^ Interestingly, the enteric nervous system expresses similar neurotransmitter as the CNS and, presumably because of it, patients with neurodegenerative disorders like Alzheimer and Parkinson patients are reported to often have neurodegenerative pathology in the gastrointestinal system as well.^11^ This might be also involved in causing neurological dysfunction and gastrointestinal symptoms in the GWI as well.

The third factor listed by Kimono^11^ was the altered microbiome. We will summarize this separately in the next section.

## 3 GUT MICROBIOME AND GWI IN HUMANS

A healthy human digestive system contains about 10^13^–10^14^ different types of bacteria, which forms the gut microbiome,^25^, ^26^ although there are large differences among literatures.^27^ We have written above that there is a bidirectional communication between the enteric nervous system and the brain. Such communication modifies hormone secretion, affects the immune system, affects the satiety, the mood, and, for example, stimulate nausea and gut movements. In addition, there is a bidirectional signalling between the gastrointestinal system and the brain mediated by the gut microbiota, which can affect the physiological condition of the whole body system.^26^, ^28^ It is reasonable to imagine, for example, that, once the body system contracts a disease or turn into malfunctioning condition because of exposures to toxic chemical compounds, and the body starts to lose the gut integrity (increase in the apoptosis, decrease in the cells turn over and so on), the healthy gut microbiota will also collapse. The bacteria in gut microbiome play a significant role in regulating the so‐called gut–brain axis in addition to the digestion and absorption of nutrients^26^, ^29^ that alteration in the gut microbiome profile and gut dysbiosis can affect the immune system, the endocrine system, brain function, other than causing alteration and/or malfunctioning in the digestive system.^26^ In addition, a problem of this change is that in such changes in the microbiota profile, the changes are from the probiotic species of bacteria that promote health to the ones that cause or increase inflammation, that is the proinflammatory type of bacteria.^11^, ^29^, ^30^

Studies have shown that various diseases cause dysbiosis and altered gut microbiota profile. For example, patients with myalgic encephalomyelitis (also known as chronic fatigue syndrome) and inflammatory bowel disease are found to have dysbiosis and reduced diversity in the species.^12^, ^31^, ^32^ Patients with inflammatory bowel disease had about 25% less microbial genes compared to healthy persons.^32^

Similarly, in patients with GWI, the gut microbiome is significantly different compared to Gulf War veterans who do not have GWI.^16^ There were also differences between the patients with GWI with and without gastrointestinal symptoms. Patients with GWI and gastrointestinal symptoms had less *Firmicutes* compared to patients with GWI without gastrointestinal symptoms and compared to healthy GW veterans.^16^ Interestingly, *Bacteroidetes* was more abundant in the patients with GWI and gastrointestinal symptoms compared to patients with GWI without gastrointestinal symptoms and healthy GW veterans.^16^ At the family level and genus level, however, there were no significant differences between the patients of GWI with and without gastrointestinal symptoms, but there were differences found between the healthy GW veterans and patients with GWI.^16^ Healthy GW veterans had significantly more *Lachnospiraceae* and less *Ruminococcaceae*, *Streptococcaceae*, and *Verrucomicrobiaceae* compared to patients with GWI with or without gastrointestinal symptoms,^16^ suggesting that such differences in the microbiota profile could be related to the GWI rather than specifically to the gastrointestinal symptoms.

So, what do these differences mean? Is it bad to have more *Bacteroidetes* and less Firmicutes? Is it bad to have less *Lachnospiraceae* and more *Ruminococcaceae*, *Streptococcaceae*, and *Verrucomicrobiaceae*? It is difficult to discuss about the microbiota profile of patients with GWI without the information at the species level because there are both beneficial species and unbeneficial species in these groups. Studies comparing the gut microbiota between the non‐obese and obese humans have shown that there are differences in the microbiota profile.^33^ Forty‐six genera were found different in the abundance, and they found that the major differences were in only nine bacteria species, some significantly more present and some significantly less present in the two groups.^33^ These nine bacteria species were *Clostridium bolteae*, *C. symbiosum*, *C. clostridioforme*, *C. ramosum*, *Ruminococcus gnavus*, which were more present in the obese group, and *Faecalibacterium prausnitzii*, *Roseburia inulinivorans*, *C. eutactus* and *Methanobrevibacter smithii*, which were more present in the non‐obese group. *F. prausnitzii* is known as an anti‐inflammatory bacteria species.^34^ *F. prausnitzii*, *R. inulinivorans* and *C. eutactus* produce butyrate,^35^ which has significant effects in intestinal homeostasis and energy metabolism, enhancing intestinal barrier function through its anti‐inflammatory properties.^36^ *M. smithii* possesses various beneficial effects on health, including its capacity to consume products of other bacterial fermentation like methanol, ethanol and ammonium.^37^ *R. gnavus* ^38^ and *C. bolteae* ^39^ are known as a proinflammatory bacteria species which synthesize and secrete inflammatory polysaccharide.^38^ The major differences in the types of bacteria species in obese and non‐obese people indicate the importance of quality differences in the gut microbiota other than the quantitative differences, and this study also shows the importance of discussing the differences in the microbiota at the species level. Studies have found that the GW veterans with obesity are more likely to have health problems.^40^ It could be that alteration in the gut microbiota is involved.

Studies showing differences in the gut microbiota at species level in the patients with inflammatory bowel disease, which is often reported in the GWI patients, have shown a depletion in the *F. prausnitzii* and *R. hominis* and increase in *Escherichia coli*.^41^ *R. torques* and *R. gnavus* were more present in the inflammatory bowel disease patients although there were differences in the abundance depending on the forms, that is the Crohn's disease or ulcerative colitis, of inflammatory bowel diseases.^41^ Butyrate density was decreased in these patients showing consistency with the depletion in the butyrate producers.^41^ This study suggests that the dysbiosis and the changes in the gut microbiota to the proinflammatory types of them are both significantly involved in causing the symptoms of inflammatory bowel diseases.

As such, it is important to consider dysbiosis and alteration in the gut microbiota at the bacteria species level, and the impacts of the decrease in the anti‐inflammatory chemicals and the increase in the proinflammatory chemicals on gut environment. The inflammation triggered by such changes will be informed to the brain through the gut–brain axis, and the whole system level changes can be initiated or facilitated by the changes in the gut. These systematic changes also suggest the existence of a negative loop, that is the disease that triggers changes in microbiota, specifically dysbiosis in the beneficial bacteria species and increase in the proinflammatory bacteria species, would negatively change the chemical environment in the gut. Through the communication of gut–brain axis, this will affect the neuroendocrine/hormone secretion in the brain and affect the immune system, and these changes would further deteriorate the gut function. How to end the loop, and at which step in this loop we should intervene to effectively end the loop and improve the conditions will be the question. Understanding of the gut–brain axis is significant and mechanistic insights into the loop might provide the possible strategies for such efforts.

## 4 ANIMAL MODELS OF GWI

### 4.1 Studies on the CNS

There are studies using animal models focusing on CNS and GWI symptoms. Experimental exposure of rats to PB, which was used to protect from nerve gas agents during the GW, and *N,N*‐diethyl‐*m*‐toluamide (DEET), which was used as insect repellent during the GW, and permethrin, a synthetic pyrethroid used as insecticide during GW, caused decrease in the hippocampal neurogenesis,^42^ caused dysfunction in spatial memory^42^ and object recognition tests,^43^ and caused an increase in the depressive‐ and anxiety‐like behaviours.^42^ In addition, they caused loss in the mature neurons, although neural stem cells in the dentate gyrus were not killed.^42^ The effects of the chemical exposure were worsened when the animals went through stress condition of 5 min restraint daily for 4 weeks.^42^ Acetylcholinesterase (AChE) activity of these animals was reduced in the midbrain, the brainstem and the cerebellum, and microtubule‐associated protein (MAP‐2) expression was reduced and glial fibrillary acidic protein (GFAP) expression was increased in the cerebral cortex and at hippocampus.^44^ High mobility group box‐1 (HMGB1), which is released when inflammation occurs,^45^ and proinflammatory cytokines were increased in the cerebral cortex and in the neuron‐derived extracellular vesicles,^43^ indicating the existence of inflammation in the brain.

In a study using mice as animal model system, sex differences in the influences of chemical exposure were found.^46^ Object recognition was more impaired in female mice exposed to GW chemicals than males, whereas plasma inflammatory cytokines were more elevated in males.^46^ Endogenous acetylcholine levels in the brain homogenates of mice exposed to chlorpyrifos or ‘chlorpyrifos + PB + permethrin’ were significantly increased in the mouse that went through chemical exposure.^47^ Neuronal precursor marker doublecortin (DCX) expression in the dentate gyrus was significantly reduced in the mice that went through chemical exposure, suggesting the reduced hippocampal neurogenesis.^47^ A thorough study testing the influences of exposure to PB, permethrin and DEET showed that chemical exposure induced cognitive dysfunction (in the tests on novel object recognition, passive avoidance and Barnes maze), depression or behavioural despair (at forced swim test), insulin insensitivity, gut dysbiosis, endotoxemia and neuroinflammation (increased hypertrophied GFAP‐positive astrocytes, interleukin‐6, neuropathic pain transcripts).^48^, ^49^ These studies showed that the cognitive dysfunction observed in the patients of GWI could be due to the reduced neurogenesis as well as apoptosis of mature neurons and the neuroinflammation.

### 4.2 Studies on the gastrointestinal system

#### 4.2.1 Influences of PB on gastrointestinal system and gut–brain‐gut loop

Compared to the studies on the CNS system, the studies on the gastrointestinal system in the GWI using animal model system started only recently. In 2017, Alhasson et al.^14^ reported that in a mouse model of GWI, exposure to PB and permethrin increased the ratio of *Firmicutes* and *Tenericutes*, compared to *Bacteroidetes*, in the samples collected from ileal and caecal segments of the intestine and in the faecal pellet samples. These results in mice are not consistent with the findings in humans, which showed less abundance of *Firmicutes*,^16^ and further studies using different animal model system and studies at bacteria species level may be necessary to discuss in detail.

The study by Alhasson et al.^14^ showed that the mice exposed to PB and permethrin had almost two‐fold increase in the blood endotoxin level, which reduced to control group level by administration of antibiotics,^14^ suggesting that the increase in the endotoxin was due to the changes in the gut microbiota and that the endotoxin can be transported to other locations in the body through the blood system. They found that the level of toll‐like receptor 4 (TLR4), which activates the nucleus factor kappa B (NF‐κB) inflammatory pathway, was increased in the frontal cortex and olfactory bulb of the brain as well as in the ileum of the intestine, and the mice administered with antibodies showed significantly reduced TLR4 level. These results indicate that the chemical exposure can cause altered gut microbiota and reduced microbiota, the dysbiosis. Inflammation at the gut caused by the exposure can cause dysbiosis^50^ but the dysbiosis can also cause inflammation.^51^ In addition, at distant locations from the gut, such as the brain, the transportation of the endotoxins by the blood circulation can cause inflammation.

Other than the vascular system to transport the endotoxin, enteric nervous system can also transport the information from the gut to the brain and initiate the gut–brain‐gut loop. Mice exposed to PB and permethrin by gavage showed increases in the stool endotoxin level, and increases in HMGB1, GFAP (co‐localized with TLR4 indicating the increase of TLR4 in the glia cells), S100 calcium binding protein B (S100B) and toll‐like receptor 2 (TLR2) mRNA expression in the small intestine tissues.^52^ HMGB1, GFAP and S100B are all glia cell markers. TLR2 is involved in ligand‐specific recognition and signalling by heterodimerization with TLR1 or TLR6, and it initiates an intracellular signalling pathway by adopting an adaptor protein MyD88.^53^ As already written above, HMGB1 is released upon inflammation activation.^45^ These results show that exposure to PB and permethrin stimulated inflammatory pathways in the enteric glia cells in the small intestine.

#### 4.2.2 The role of extracellular vesicles in cell–cell communication and gut–brain axis

The third communication tool from the gut to the brain most recently discovered is the EV. EVs are phospholipid membrane enclosed vesicles secreted from prokaryotes to eukaryotes, that is from mammalian cells, plant cells and bacteria. They are similar especially to retroviruses: (1) in the size, (2) in the biogenesis, (3) the lipid membrane structure, (4) the possibilities that they can bind to cells and enter them through fusion or endocytosis, (5) the fact that they trigger specific reactions from these cells, and (6) the fact that the genetic materials they carry can affect the cells they entered, but they do not replicate like viruses.^54^ Some studies even describe that they are almost like a virus that do not replicate.^54^

Although the finding of the existence of EV goes back to as early as in the 1940s, they were first considered debris or ‘platelet dust’ without biological functions.^55^ Following the much recent finding that they contain RNA, they started to receive strong interests and the studies on the possible biological function of EVs on cell‐to‐cell communication have increased.^55^ There are three types of EVs, that is the apoptotic bodies, the microvesicles or the ectosomes, and the exosomes, different in their sizes and biogenesis.^55^ They contain lipids, proteins and nucleic acids, and the compositions depend on the type of cells they originated.

Considering that EVs are synthesized and released from various types of cells, it is possible to consider that, in the gut, there are EVs from the bacteria and from the intestinal epithelium. So far, there have been no methods developed yet to separate the EVs from bacteria and EVs generated by the intestinal epithelium.^56,^, * How the EVs from the bacteria and the intestinal epithelium cells of the host interact and affect each other are mostly not known yet.

Extracellular vesicles can both promote diseases and suppress diseases^54^ through the intercellular communication mechanisms. The intestinal epithelium cells produce EVs and these EVs of intestinal epithelium origin are found to suppress inflammatory bowel disease severity.^57^, ^58^ In vitro studies have shown that the EVs from large intestine epithelial cells suppressed the proliferation of CD4+ immune cells.^57^ Injection of EV from intestine of mice with experimentally induced sepsis at the caecal intestine to other mice with dextran sulfate sodium induced colitis made a significant reduction in the proinflammatory cytokines, tumour necrosis factor alpha (TNF‐α) and IL‐17a, compared to the mice that did not receive EVs.^58^ The intestinal epithelial cells produce EV with transforming growth factor beta (TGF‐β),^57^ which has anti‐inflammatory effects as well as maintaining T‐cell homeostasis and regulation of T‐cell function.^59^ These studies suggest that the EVs from the intestinal epithelial cells might be beneficial in therapeutic use because of the effects to suppress inflammation.

Interestingly, studies using the EV from the plasma of septic mice enhanced the level of proinflammatory cytokines in vitro and neutrophils and macrophages in vivo.^60^ This increase was not observed in the TLR7 knockout mice and MyD88 knockout mice,^60^ which suggested that TLR7‐MyD88 signalling is involved in the reaction to the administration of plasma EV.

These differences in the effects of EV could be due to the differences in the molecules that the EV carries depending on the cells they originated and the environmental change/exposure. Major molecules found so far that EV carries and considered to be involved in the inter‐cell communication are annexin‐A1 (ANXA1) and microRNAs other than TGFß.

ANXA1 is known with the anti‐inflammatory properties and the facilitation of mucosal wound healing.^61^ Following intestinal injury, and in inflammatory bowel disease patients, the intestinal epithelium cells and immune cells produce EVs that carry ANXA1 and activate G‐protein coupled formyl peptide receptor 1 (FPR1) and 2 (ALX/FPR2).^61^ This initiates and facilitates the wound closure. There are several mechanisms suggested. One is through the suppression of proinflammatory mediators, another is through accelerating apoptosis by activating caspase‐3 activity and/or increasing cytosolic calcium influx and activating the apoptotic system.^62^

MicroRNAs are noncoding RNA fragments. They can be expressed in the EVs and affect gene expression in the recipient cells of EV.^63^ They are involved in cell cycle control, cell differentiation, apoptosis and cytokine expression.^63^ MicroRNA can bind to the TLR receptors expressed in immune cells, activate NF‐κB signalling, and increase the secretion of IL‐6 and TNFα, thus stimulating inflammation. Studies have found that microRNA‐21 is overexpressed when there is intestinal inflammation and that elimination of microRNA‐21 using transgenic mice enhances the survival rate of mice with experimentally induced colitis by protecting from inflammation and tissue damage.^64^ Another study has shown as well that the transgenic mice without microRNA‐21 (miR‐21^−/−^ mice) were less susceptible to dextran sodium sulphate induced colitis than the wild‐type mice.^65^ These studies suggest the proinflammatory effects of microRNA‐21. Knockout of microRNA‐21 also suppressed biliary hyperplasia and the development of liver fibrosis in the mice with bile duct ligation.^66^

These studies show that the EVs have significant roles in the gut environment, and the roles can be both positive influences and negative depending on the molecules they carry.

### 4.3 Changes in the gut microbiota and virome

Exposure to Gulf War chemicals has been shown to change the gut microbiome and virome.^14^, ^16^, ^17^, ^66^ The gut microbiome showed dysbiosis and viral richness in the mice exposed to PB and permethrin by oral gavage.^67^ There was alteration in the types as well, that is decrease in the *Microviridae* bacteriophages and increase in the *Siphoviridae* and *Myoviridae* in the mice exposed to PB and permethrin.^67^ The mice exposed to GW chemicals had *Firmicutes* and *Bacteroidetes* as the dominant species in their bacteria microbiome, which became reduced to the control group level by antibiotic treatment.^67^ The influence of GW chemicals on the increase of *Firmicutes* was found in other studies using mice as animal model^14^ and in human studies,^16^ although the results on *Bacterioidetes* were not consistent.^14^ The GWI mouse models showed increases in gram negative bacteria *Allobaculum*, *Coprococcus* (Phylum *Firicutes*), *Dorea* (Phylum *Firicutes*), *Turibacter* and *Ruminococcus*.^14^ As mentioned earlier, there are species that are beneficial and non‐beneficial in the same genus or family. For example, *Ruminococcus gnavus* is known to have correlation with the symptoms of inflammatory bowel diseases,^38^, ^68^ although, in general, the *Ruinococcus* genus possess an important role in degrading polysaccharides into various nutrients, that is cellulolytic, and found in abundance often in ruminants.^69^ On the contrary, *Akkermansia muciniphila* is significantly decreased in the animal model of GWI.^17^ *Akkermansia muciniphila* is one of the bacteria species that is abundant in human gut with probiotic function and contributes in degrading mucins.^70^ Recent studies have shown that the EV from *A. muciniphila* can reduce gut barrier disruption by enhancing the tight junction function and improving glucose tolerance in high‐fat diet induced type two diabetes.^71^, ^72^

These studies suggest that providing supplements that can increase probiotic bacteria may improve the gut microbiota and improve the GWI symptoms. Giving mice butyrate during exposure with PB and permethrin resulted in increased butyrogenic bacteria, and decreased TLR4 activation.^73^ Lacto‐N‐fucopentaose‐III is a glycan in human milk and also increased butyrate producer bacteria such as *Butyricoccus* and *Ruminococcous* in mice exposed to GW chemicals.^74^ These treatments to mediate butyrate producing bacteria resulted in improved gut health and decreased inflammation.

## 5 DIET AND GUT DYSBIOSIS

Other than supplements, foods themselves can affect gut microbiota. Gut microbiota obtains nutrients from the diet (including liquids) that the host takes.^27^ What we eat and drink affects the microbiota and virome profile. This indicates that the diet has the possibility to improve and worsen the microbiota and virome profile. There are various phytochemicals with bioactive properties of, for example, anti‐inflammation.^75^, ^76^, ^77^ By choosing the types of foods, it is possible to take higher amount of phytochemicals with the possibilities to heal diseases.^78^, ^79^ Recent study has shown that curcumin treatment along with exposure to GW chemicals showed better cognitive function and mood in the rats.^80^ These rats showed reduced neuroinflammation in the brain as well.^80^ Although the study did not examine gut microbiota, there is a high possibility that the improvement in the brain took place by first improving the gut system and the improvement at the gut generated the improvement in the brain by the gut–brain axis communication. Indeed, although not from the focus on GWI, recent studies have shown that curcumin administration had similar effects as type 2 diabetes medication metformin on attenuating the fat hepatic ectopic fat deposit and reversed the negative influences of high‐fat diet on gut microbiota.^81^ Administration of nanoparticle curcumin with enhanced bioavailability suppressed the influences of experimentally induced colitis by increasing butyrate producing bacteria.^82^ Studies have discussed its effects on promoting beneficial bacterial strains, improving intestinal barrier functions.^83^ Thus, it is highly likely that the treatment using curcumin would have beneficial effects on GWI through its effects on improving gut microbiota. There are also other phytochemicals with anti‐inflammatory effects shown to have positive influences on restoring gut microbiota in mice with experimental colitis (e.g. kaempferol,^84^ black current^85^ and ß‐caryophyllene^86^). ß‐caryophyllene is known as a phytochemical that activates cannabinoid receptor 2 (CB2) and activation of CB2 has anti‐inflammatory effects.^87^, ^88^ Recent studies have shown that exposure to ß‐caryophyllene down‐regulates the genes related to inflammation and up‐regulates these related to cell proliferation.^76^ These studies suggest that utilization of foods with high amounts of phytochemicals with anti‐inflammatory effects may produce positive effects on GWI.

In contrast, there are many foods that can worsen the symptoms. One of these groups are the ‘high fat diet’.

### 5.1 High‐fat diet

The majority of the Gulf War veterans are considered overweight or obese. High‐fat diet can affect the gut microbiota profile and cause dysbiosis, which also links to inflammatory bowel diseases and obesity.^89^ The low‐grade gut inflammation as a result of a high‐fat diet may affect the severity of GWI. Studies have shown that mice exposed to PB and permethrin in addition to being fed with high‐fat diet resulted in a significant decrease in the amount of butyrogenic bacterial species, such as *Lachnospiraceae bacterium*, *Eubacterium* species, *Ruminococcacae*, *Enterorhabdus* species and *Hungatella*.^40^ Proinflammatory cytokine IL‐6 level was high in the GWI mice, and these fed with high‐fat diet showed significantly worsened, that is even higher level of IL‐6, compared to the GWI mice fed with regular chow.^40^ High‐fat diet itself, without GWI, affected the mice to have higher proinflammatory cytokine IL‐1β level, and mice exposed with GW chemicals showed even higher level of IL‐1β.^40^ These results show that high‐fat diet and obesity it causes have negative influences on mice with and without GW exposure. In another study, similarly to the study by Bose et al.,^40^ GW chemical exposure altered gut microbiota profile and the feeding of high‐fat diet had worsened it, and, interestingly, returning the food to regular chow improved the gut microbiota to control level.^90^ These are promising results, indicating that changes in the diet can improve the GWI symptoms.

## 6 CONCLUSION: POSSIBLE NEW STRATEGIES ON THE TREATMENT FOR GWI

**FIGURE 2:**
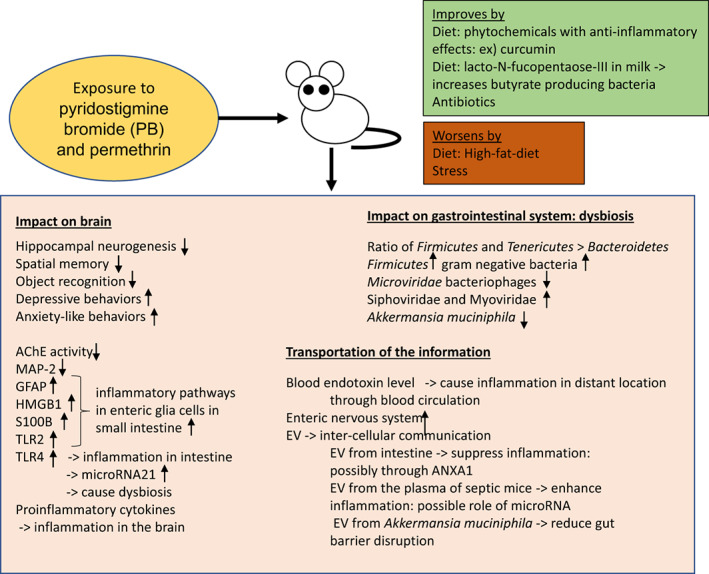
Summary of Gulf War Illness (GWI) mouse studies. Studies using animal models showing the causation of the symptoms of GWI are also accumulating. Exposure to gulf war chemicals resulted in damage to nervous systems and immune systems, including neuroendocrine and immune dysregulation, autonomic nervous system irregularities, and reduced white and grey matter in brains.

As summarized here, there are new studies using animal models starting to show the bacteria and virome that are changed by the GW chemicals, and there are factors that are shown to negatively affect or improve the conditions (Table 1). Studies using animal models showing the causation of the symptoms of GWI are also accumulating (Figure 2, Table 2). The new studies showing promising results to improve the GWI symptoms can be the new directions in treating GWI: the intestine EVs,^57^ utilization of the phytochemicals with anti‐inflammatory effects^77^, ^80^, ^85^, ^91^ and reducing the intake of high‐fat diet.^90^ All of these have shown positive results in animal model studies as shown above.

**TABLE 1:** Summary of the examples of the factors discussed to have influence on gut microbiota, cytokines and brain function in animal models

Negative/positive	Factors that affect	Chemical compounds involved	Effects	Bacteria affected	References
Negative	GW chemicals	PB, permethrin, DEET, chlorpyrifos	Dysbiosis Alteration of gut microbiota Alteration of virome Causes neuroinflammation Increase of stool endotoxin Increase in proinflammatory cytokines Increase in gut permeability Increase in exercise intolerance Spatial memory dysfunction Reduced object recognition Reduced hippocampal neurogenesis Depression/anxiety Insulin insensitivity	Virome: decrease in the *Microviridae*, and increase in *Siphoviridae*, *Myoviridae* Bacteria: *Firmicutes* and *Bacteroidetes* dominant species; increases in *Allobaculum*, *Coprococcus*, *Dorea*, *Turibacter*, *Ruminococcus*; *Akkermansia muciniphila* decreased	14, 17, 40, 42, 43, 44, 46, 47, 48, 49, 67, 73, 74, 80, 90, 92, 93
	High‐fat‐diet, obesity		Obesity affects gut microbiota profile	Decrease in *Lachnospiraceae bacterium*, *Eubacterium* species, *Ruminococcacae*, *Enterorhabdus* species and *Hungatella*	40, 90
	Stress/restraints corticosterone		Corticosterone or restraints were often used together with exposure to GW chemicals; none or few are conducted as separate factor in GW studies		42, 43, 44, 48, 49, 73, 80
Positive	Butyrate	Butyrate	Increases over fivefold in the abundance of butyrate producing bacteria *Lactobacillus*, *Roseburia* and *Bifidobacterium*		73
	Phytochemicals	Black currant Curcumin Kaempferol Beta‐caryophyllene	Curcumin: improved cognitive function, suppressed NF‐kB activation in colonic epithelial cells, suppressed development of colitis, increased butyric producing bacteria *Clostridium IV* and *Clostridium XIVa* Kaempferol: elevated *Firmicutes* to *Bacteroidetes* ratio, increased *Prevotellaceae*, *Ruminococcaceae*, and reduced *Proteobacteria* ß‐caryophyllene: reduced severity of colitis, reduced colonic damage, inhibited activation of NF‐kB and suppressed inflammatory cytokines level		80, 81, 82, 83, 84, 85, 86
	EV	Intestine EV	Large intestine EV suppressed CD4+ immune cells; injection of EV suppressed proinflammatory cytokines in other mice with colitis; microRNA, TGFß and ANXA1 possibly involved in the effects of EV; septic EV injection reduced proinflammatory cytokines TNFα and IL‐17a		57, 58
	Lacto‐N‐fucopentaose‐III	Lacto‐N‐fucopentaose‐III	Glycan in human milk increased butyrate producing bacteria	*Butyricoccus* and *Ruminococcous* increased	74

Abbreviations: ANXA1, annexin A1; DEET, *N,N*‐diethyl‐*m*‐toluamide; EV, extracellular vesicles; NF‐kB, nuclear factor kappa B; PB, pyridostigmine bromide.

**TABLE 2:** Animal studies on the influences of GW chemicals summarized along the years of publication

	Animals used	GW chemicals used	Amount used	Routes	Results	References
Parihar et al. (2013)	Rats	PB, DEET, PE	1.3, 40, 0.13 mg/kg, respectively	Gavage, topical, topical, respectively	Exposure to GW chemicals caused depressive and anxiety‐like behaviours, impaired spatial memory in Morris water maze, and reduced hippocampal neurogenesis; stress by being restrained 4 min daily for 4 weeks worsened the symptoms	42
Ojo et al. (2014)	Mouse	PB, PE, Chl	0.7, 200, 5 mg/kg	IP	GW chemicals caused increase in acetylcholine levels, decrease in doublecortin levels and increase in GFAP. Platelet endothelial cell adhesion molecule was suppressed, and intercellular adhesion molecule expression was increased	47
Alhasson et al. (2017)	Mouse	PB, PE	2, 200 mg/kg	Gavage	GW chemicals caused dysbiosis and altered microbiota; caused neuroinflammation	14
Seth et al. (2018)	Mouse	PB, PE	2, 200 mg/kg	Gavage	GW chemicals caused dysbiosis; administration of butyrate to GW mice improved the gut microbiota profile, reduced a leaky gut marker Claudin‐2 expression, and reduced TLR4 and TLR5 expression in the liver; butyrate administration to GW mice also increased the expression of GPR109A (also called HCA2), which has anti‐inflammatory effects,^94^, ^95^ protein in the villi of intestine	73
Kodali et al. (2018)	Rat	PB, PE, DEET	1.3, 0.13, 40 mg/kg	Gavage, topical, topical	Exposure to GW chemicals suppressed hippocampal neurogenesis and cognitive function; administration of curcumin improved the hippocampal neurogenesis and cognitive function to control group level; curcumin administration reduced the astrocyte hypertrophy and activated microglia to control level	80
Seth et al. (2019)	Mouse	PB, PE	2, 200 mg/kg	Gavage	GW exposure altered enteric virome, especially of bacteriophages, and the increase of viral richness positively correlated with gut bacteria dysbiosis and proinflammatory cytokine level; GW exposure also caused weakening of intestinal tight junction; IL‐6, IL‐1ß, IFN‐, and Claudin‐2 were increased, and BDNF was decreased	67
Madhu et al. (2019)	Rat	PB, DEET, PE	2, 60, 0.2 mg/kg	Gavage, topical, topical	HMGB1, TNFα, IL‐1ß, C3 and TccC5b‐9 expression were higher in the GW mice; the patterns of expression of HMGB1 were altered; GFAP level was higher (astrocytes hypertrophy), and IBA1 and ED1 were more expressed (more microglia)	43
Kimono et al. (2019)	Mouse	PB, PE	2, 200 mg/kg	Gavage	GW exposure caused increases in the bacterial endotoxin in stools, HMGB‐1 and GFAP in small intestine, TLR2, TLR4, TLR5, S100B and RAGE in the enteric glial cells in the small intestine by quantification of colocalization with GFAP; activation of NADPH oxidase 2 (NOX‐2) in small intestine; the results suggested the activation of TLR4‐S100ß/RAGE‐iNOS pathway	52
Hernandez et al. (2019)	Mouse	PB	9 μg/ml or 90 μg/ml	Drinking water for 1 week	PB made changes in gut motor function; sex differences in the results of chemical exposure, increasing neurogenic contractions in males and decreased them in females; PB did not affect gut permeability	92
Angoa‐Perez et al. (2020)	Mouse	PB, PE	0.7, 200 mg/kg	IP, IP	Altered microbiota; HFD worsens the symptoms, change of diet to regular chow returned the levels to control level	90
Bose et al. (2020)	Mouse	PB, PE	2, 200 mg/kg	Gavage	Altered microbiome and abundance; obesity worsens	40
Hernandez et al. (2020)	Mouse	PB, PEA	90 μg/ml; 0.07 mg/ml	In drinking water	By administering separately and both, PE alone was found sufficient to affect. Glial reactivity and changes in enteric neurochemicals; sex differences in the effects were found; PB alters the effects of PEA; PEA suppressed colonic transit in male mice; PB alone did not cause changes in the colonic cytokines/chemokines' expression; PE increased colonic proinflammatory cytokines/chemokines	93
Kimono et al. (2020)	Mouse	PE, PE	2, 200 mg/kg	Gavage	Exposure to GW chemicals caused dysbiosis and increase in HMGB1 levels in serum; *Akkermansia muciniphila* was significantly decreased in GW mice; Claudin 5 mRNA levels at BBB endothelial cells of blood vessels, determined by colocalization with CD31, were significantly decreased in the frontal cortex; instead, microglial markers HMGB1, TMEM119, NLRP3, 3‐nitrotyrosines, IL‐6, IL‐1ß and IL‐18 were significantly increased in the frontal cortex suggesting neuroinflammation in the brain	17
Mote et al. (2020)	Mouse	PB, PE	0.7, 200 mg/kg	IP	GW exposure did not cause alterations in gut microbiota profiles, but gut motility was slower and faecal lipocalin 2 level was higher in the GW mice; Lacto‐N‐fucopentaose‐III (LNFPIII) administration improved the gut motility and lipocalin 2 level of GW mice	74
Appiah et al. (2020)	Mouse					58
Bryant et al. (2021)	Mouse	PB, PE	0.7, 200 mg/kg	IP	Sex differences in the results; object location memory was more impaired in females; females showed more astrocyte hypertrophy (GFAP‐positive cells) and activated microglia (IBA‐1 positive cells); cytokine expression was increased in females than males in hippocampus	46
Kozlova et al. (2022)	Mouse	PB, PE, DEET	6.5 mg/kg or 8.7, 1.3 mg/kg, 33% in EtOH	Gavage, topical, topical	IL‐6 level significantly elevated in mice exposed to higher amount of PB; GFAP expression at the CA1 area of hippocampus higher in the mice exposed to both lower and higher dose of PB; higher dose of PB caused alteration in gut microbiota	48
Kozlova et al. (2022)	Mouse	PB, PE, DEET	6.5 mg/kg or 8.7, 1.3 mg/kg, 33% in EtOH	Gavage, topical, topical	Mice exposed to higher amount of PB showed exercise intolerance, slower fear‐conditioning, but lower dose of PB caused higher rate of despair like behaviour	49

Abbreviations: Chl, chlorpyrifos; DEET, *N*,*N*‐diethyl‐meta‐toluamide; IP, intraperitoneal; PB, pyridostigmine bromide; PE, permethrin; PEA, palmitoylethanolamide.

**FIGURE 3:**
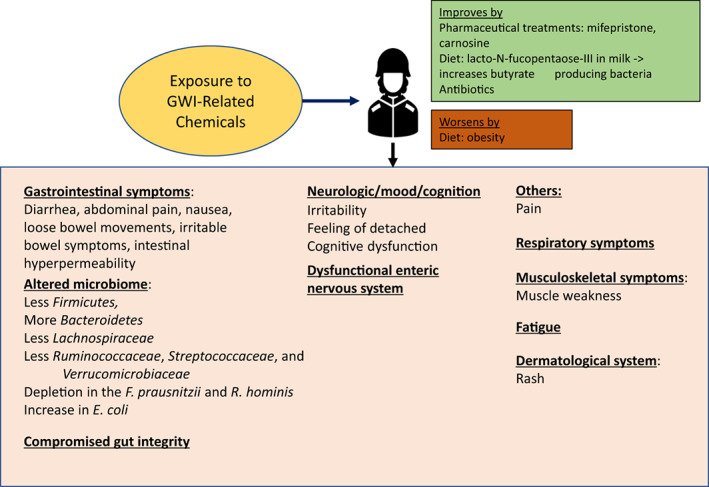
Summary of Gulf War Illness (GWI) human studies. GWI patients also suffer from a multi‐system disorder characterized by fatigue, joint and muscle pain, headaches, concentration and memory problems, gastrointestinal distress and skin rashes. In clinical situation, changes in the types of diet can be easy for some people patients but hard for others. The development of supplements that contain various phytochemicals with anti‐inflammatory effects^77^, ^80^, ^85^ or milk glycan that improves gut microbiota profile^74^ may help reducing the symptoms and/or can ameliorate the impacts of high‐fat diet. Many studies have been done on the potential pharmaceutical treatments for GWI.

Figure 3 shows the summary of the human studies we referred. In clinical situation, changes in the types of diet can be easy for some patients but hard for others. The development of supplements that contain various phytochemicals with anti‐inflammatory effects^77^, ^80^, ^85^ or milk glycan that improves gut microbiota profile^74^ may help reducing the symptoms and/or ameliorate the impacts of high‐fat diet. Many studies have been done on the potential pharmaceutical treatments for GWI. These include studies on mifepristone^96^, ^97^ and carnosine,^97^ which had some benefits. Utilization of the biologic chemical compounds that are in our system (EV) or in the healthy type of diet can become a new strategy with less risk of side effects because of their biologic nature. From the same reason, it also has possibilities to use in addition to medical treatments. The new research about the altered gut microbiome is opening up a new direction for researchers to look at for the development of novel treatments for GWI.

## AUTHOR CONTRIBUTIONS

**Elise Slevin:** Data curation (equal); writing – original draft (equal); writing – review and editing (equal). **Sachiko Koyama:** Conceptualization (equal); data curation (equal); writing – original draft (equal); writing – review and editing (equal). **Kelly Harrison:** Conceptualization (equal); writing – original draft (equal). **Ying Wan:** Conceptualization (equal); writing – original draft (equal); writing – review and editing (equal). **James E. Klaunig:** Conceptualization (equal); validation (equal). **Chaodong Wu:** Conceptualization (equal); project administration (equal); validation (equal). **Ashok K. Shetty:** Project administration (equal); supervision (equal); writing – review and editing (equal). **Fanyin Meng:** Conceptualization (lead); funding acquisition (lead); project administration (lead); writing – original draft (lead).

## FUNDING INFORMATION

The work was supported by the VA Merit Review Award to Dr. Meng (1I01BX001724) from the United States Department of Veteran's Affairs Biomedical Laboratory Research and Development Service, U.S. National Institutes of Health (NIH) National Institute of Diabetes and Digestive and Kidney Diseases Grants DK054811, DK076898 and DK107310 to Dr. Meng, NIH National Institute on Alcohol Abuse and Alcoholism Grants AA025997 and AA025157 to Dr. Meng, and a Nature Science Foundation of China (No. 81873563) and Sichuan Science and Technology Program (2022YFS0614) to YW; the project described was supported by the Indiana University Health—Indiana University School of Medicine Strategic Research Initiative. Dr. Meng acknowledges the support from PSC Partners Seeking a Cure. This material is the result of work supported by resources at Richard L. Roudebush VA Medical Center, Indianapolis, IN. The views expressed in this article are those of the authors and do not necessarily represent the views of the Department of Veterans Affairs.

## CONFLICT OF INTEREST

The authors have declared that no conflict of interest exists.

## Data Availability

Data sharing not applicable to this article as no datasets were generated or analysed during the current study.

## Endnote

* Intestinal epithelium cell adhesion molecules (EpCAM) and tetraspanins, especially CD9 and CD63, are expressed in intestinal epithelial cells' EV that some studies utilize this as a marker to determine the nature of the EV collected.^57^
